# Human-centered AI as a framework guiding the development of image-based diagnostic tools in oncology: a systematic review

**DOI:** 10.1016/j.esmorw.2024.100077

**Published:** 2024-10-07

**Authors:** K. Allen, A.K. Yawson, S. Haggenmüller, J.N. Kather, T.J. Brinker

**Affiliations:** 1 Digital Biomarkers for Oncology Group, German Cancer Research Center (DKFZ), INF 223, Heidelberg, Germany; 2 Else Kroener Fresenius Center for Digital Health, Medical Faculty Carl Gustav Carus, Technical University Dresden, Dresden, Germany; 3 Department of Medicine I University Hospital and Faculty of Medicine Carl Gustav Carus Technische Universität Dresden, Dresden, Germany; 4 Department of Medical Oncology, National Center for Tumor Diseases (NCT), University Hospital Heidelberg, Heidelberg, Germany

**Keywords:** human-centered AI, human-AI interaction, oncology, dermatology, explainable AI

## Abstract

**Background:**

Artificial intelligence diagnostic tools (AIDTs) in oncology show high image classification accuracy but limited clinical adoption. Their adoption could be enhanced by (i) using user feedback during the software design, (ii) demonstrating that AIDTs improve the user’s decisions, and (iii) providing explanations of AI decisions tailored to the user, three aspects central to human-centered AI (HCAI). This review assesses these three aspects in AIDTs for oncology in general, exemplifying its concepts in the established field of skin cancer diagnostics as a specific use case.

**Materials and methods:**

We carried out three Preferred Reporting Items for Systematic reviews and Meta-Analyses (PRISMA) searches using PubMed and ScienceDirect, limiting the results to articles published from 2019 to 2024. The first search focused on articles that used user feedback to develop AIDTs. The second search addressed whether AIDT improves dermatologists’ decisions. The third search targeted explainable AI in skin cancer.

**Results:**

Five studies incorporated user feedback in AIDT design for cancer. Zooming in on AIDT for skin cancer, nine studies (3/37 in 2019, 3/93 in 2023) indicated that AIDTs improve dermatologists’ decisions in experimental (*n* = 5) and clinical settings (*n* = 1). Explainable AI was common in skin cancer diagnostics (*n* = 26), with papers assessing the user’s preference for explainable AI (XAI) methods or the impact of XAI on the user’s trust in AI diagnosis.

**Conclusions:**

User feedback has been used to develop AIDTs tailored to clinicians’ needs. Evidence shows that AIDTs can improve clinicians’ decisions. This, combined with XAI, increases clinicians’ trust in AIDTs, potentially favoring their widespread usage.

## Highlights

• HCAI prioritizes user values, needs, and concerns in AI development.
• These principles enhance clinician decision making, as shown in different settings.
• In skin cancer, explainable AI methods have been explicitly shown to boost user trust in AI diagnoses.
• Combining user feedback, decision improvement, and XAI fosters adoption in clinical practice.

## Introduction

Artificial intelligence diagnostic tools (AIDTs) for oncology have demonstrated remarkable capabilities, such as classifying medical images with accuracies that often surpass that of humans. For instance, AIDTs can identify malignant melanoma, the most aggressive form of skin cancer, with accuracies often exceeding those of trained dermatologists.^1^, ^2^, ^3^, ^4^, ^5^, ^6^ Similarly, promising results were observed for detecting other malignant conditions, including breast, lung, prostate, and brain cancers.^7^, ^8^, ^9^, ^10^, ^11^, ^12^

Despite numerous reports of AIDTs detecting malignant tumors at expert levels in nonclinical settings, these diagnostic assistants face several challenges in clinical settings.^12^ For instance, AIDTs typically show lower accuracy when confronted with real-world data^13^, ^14^, ^15^, ^16^ due to several factors, including distribution shifts,^17^ performance based on shortcuts rather than signal,^18^^,^^19^ and model biases by factors such as ethnicity^20^, ^21^, ^22^ and gender.^23^ Additional challenges relate to the interactions between AI diagnostic assistants and clinicians.^24^, ^25^, ^26^ To be relevant in clinical settings, AIDTs must facilitate the clinician’s diagnostic workflow^27^ and be trustworthy, reliable, and safe. For instance, when faced with a diagnosis made by models with low levels of interpretability, clinicians are often hesitant to base high-stakes clinical decisions without further assurances that the AI system works as intended.^28^

**Figure 1:**
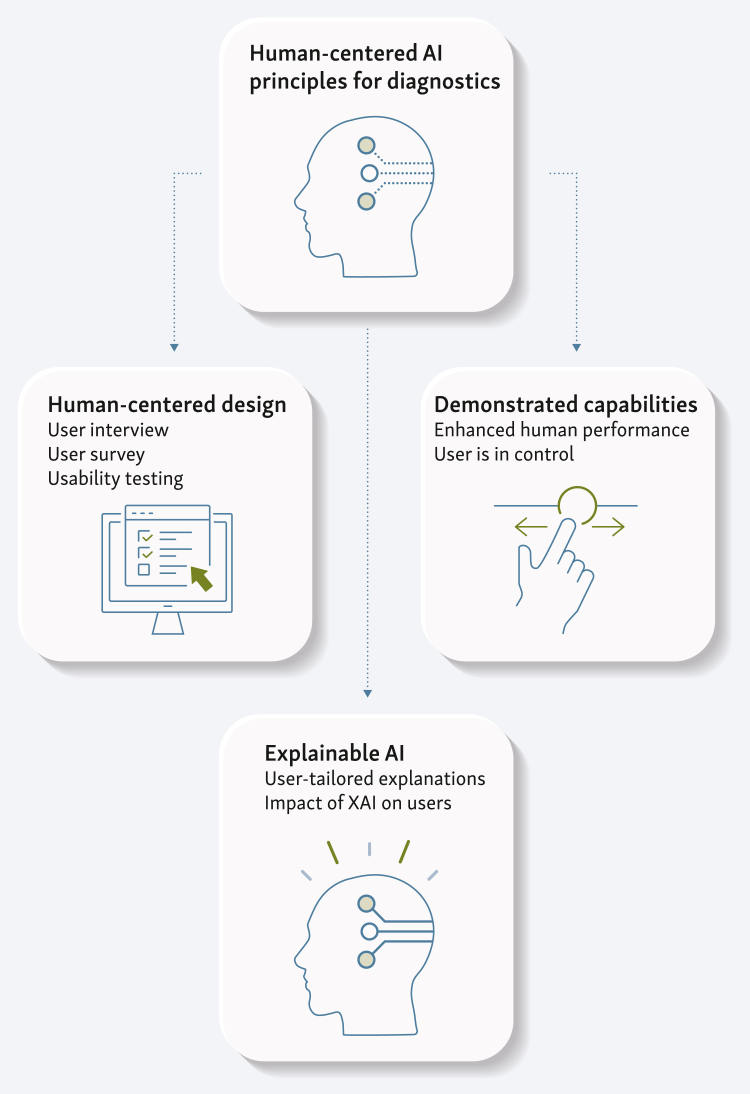
HCAI principles that can apply to the development of artificial intelligence (AI) diagnostic tools. XAI, explainable artificial intelligence.

One approach that focuses on users’ concerns is human-centered AI (HCAI).^29^, ^30^, ^31^ HCAI is an approach to AI that prioritizes human values, needs, and concerns throughout the development and deployment of AI systems. The approach encompasses many issues, from ethical considerations to more practical topics such as usability, safety, and reliability. HCAI applies the principles of human-centered design^32^ to the development of AI, which focuses on understanding the user’s needs, behavior, and experiences, as well as engaging users throughout the design process to ensure that products are easy to use and accessible (ISO 9241-210:2019). HCAI also emphasizes developing AI systems that augment human abilities and support informed decision making.^31^ To empower users, AI systems should provide information to understand, interpret, and challenge AI decisions. Explainable AI,^33^, ^34^, ^35^, ^36^ which aims to make decisions about AI systems that are understandable to the user,^37^ is, therefore, a key component of HCAI. Figure 1 presents some of the HCAI principles that apply to AIDTs.

In this systematic review, we examined articles that utilized human-centered design to ensure AIDTs for oncology address the needs and concerns of clinicians. Using dermatology and skin cancer as an exemplary case study, we found strong evidence that AIDTs improve dermatologists’ diagnostic accuracy of skin cancer. Finally, we discuss how explainable AI (XAI) can be tailored to the user and how XAI affects users’ interactions with AIDTs. Integrating human-centered approaches into AI development is crucial for bridging the gap between technological capabilities and practical, trusted clinical applications and ultimately enhancing patient outcomes in oncology.

## Materials and methods

### Search strategy

**Figure 2:**
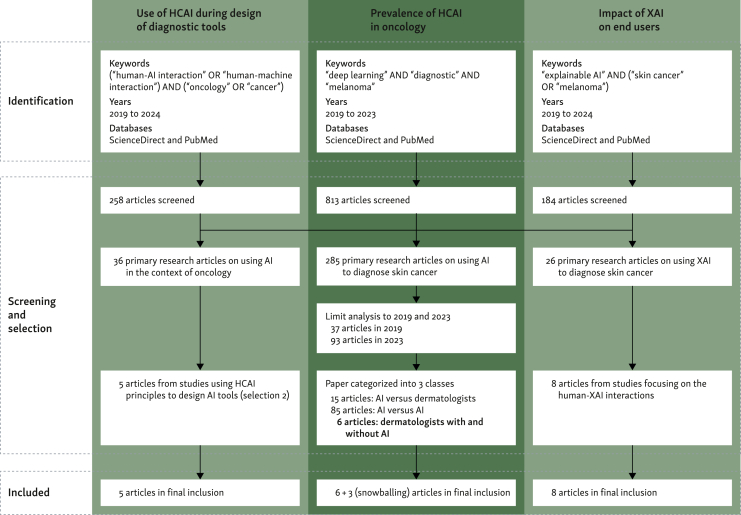
Preferred Reporting Items for Systematic reviews and Meta-Analyses (PRISMA) flow diagram of the article selection process for the three searches carried out. AI, artificial intelligence; HCAI, human-centered artificial intelligence; XAI, explainable artificial intelligence.

Using the PubMed and ScienceDirect search engines and following Preferred Reporting Items for Systematic reviews and Meta-Analyses (PRISMA) guidelines, we carried out three searches focusing on original research articles between 2019 and 2024 (Figure 2). Two scientists (KA and AKY) independently reviewed the retrieved articles to make the final selection. Articles for which the two scientists’ inclusion decisions diverged were thoroughly reviewed and discussed until a consensus was reached.

### Study selection

**Search 1**: We used the keywords ‘human-AI interaction’, ‘human-centered AI’, ‘human-machine interaction’, and ‘cancer’ or ‘oncology’, identifying 258 articles. Among these, 36 articles addressed AI applications in oncology. Our final selection included five articles that used HCAI principles during the design phase of the AI tools (Table 1).^38^, ^39^, ^40^, ^41^, ^42^

**Table 1:** Research articles using HCAI principles at the initial development phase

Authors	Year	Title	Cancer/diagnostic tool	HCAI principles	Tool developed
Xie et al.^38^	2020	CheXplain: enabling physicians to explore and understand data-driven, AI-enabled medical imaging analysis	Chest X-ray	• Survey clinicians to explore whether, when, and what kinds of explanations are needed. • Iterative software development.	CheXplain
Zhang et al.^39^	2023	PathNarratives: data annotation for pathological human-AI collaborative diagnosis	Colorectal pathological dataset	• Human–AI collaborative pathological diagnosis • Trust and confidence in AI by pathologists when AI provides detailed explanations.	PathNarratives
Calisto et al.^40^	2022	BreastScreening-AI: evaluating medical intelligent agents for human-AI interactions	Breast cancer	• Compare clinician-only versus clinician–AI. • Study clinician acceptance, expectations, and satisfaction.	BreastScreening
Calisto et al.^42^	2021	Introduction of human-centric AI assistant to aid radiologists for multimodal breast image classification	Breast cancer	• User study with 45 physicians to identify user’s needs.	BreastScreening
Lombardi et al.^41^	2023	A human-interpretable machine learning pipeline based on ultrasound to support leiomyosarcoma diagnosis	Leiomyosarcoma	• End users involved in the definition of the AI task and evaluation of the approach. • Clinicians get an understanding of the decision-making mechanisms.	Preoperative leiomyosarcoma diagnosis

AI, artificial intelligence; HCAI, human-centered artificial intelligence.

**Search 2**: We assessed the degree to which AIDTs for skin cancer were developed with an HCAI perspective. We carried out a search using the terms ‘deep learning’, ‘diagnostic’, and ‘melanoma’ for 2019 and 2023, identifying papers in which AIDTs were used to classify skin lesions. Two separate years were selected to detect potential changes over time. We found 37 relevant research articles in 2019 and 93 in 2023, respectively (Table 2). We classified these articles based on whether they focused primarily on (i) comparing the performance of AIDTs with that of dermatologists, (ii) comparing AIDTs with other AIDTs, or (iii) comparing the performance of dermatologists with and without AIDTs (final inclusion: 9 articles^28^^,^^43^, ^44^, ^45^, ^46^, ^47^, ^48^, ^49^, ^50^).

**Table 2:** Principal types of comparison carried out in research articles assessing AI-diagnostic tools for the diagnosis of melanoma

Year	Number of papers	AI versus dermatologists, *n*	AI versus AI, *n*	Dermatologists versus dermatologists + AI, *n*	Other, *n*
2019	37	8	19	3	7
2023	93	7	66	3	17

AI, artificial intelligence.

**Search 3**: We searched for articles investigating AI–human interactions in the context of XAI. As XAI techniques are becoming more commonly used, we limited our search to articles on XAI in the context of AIDTs for skin cancer. We found 26 research articles,^28^^,^^45^^,^^48^^,^^49^^,^^51^, ^52^, ^53^, ^54^, ^55^, ^56^, ^57^, ^58^, ^59^, ^60^, ^61^, ^62^, ^63^, ^64^, ^65^, ^66^, ^67^, ^68^, ^69^, ^70^, ^71^, ^72^ of which 8 focused on XAI–human interactions.^28^^,^^49^^,^^54^^,^^57^^,^^66^^,^^69^^,^^71^^,^^72^

## Results

### Designing AIDTs in oncology with a human-centered design perspective

The development of AIDTs in the framework of HCAI often relies on tools and principles of human-centered design^32^ and user experience design.^29^ At an early stage, designers interact with the users to understand their workflow, challenges, and needs. The designer typically identifies a problem or improvement opportunity that their product will address. Several user experience design research tools can be used, including user demographic research, interviews, usability testing, and analytics. The design development process typically involves creating several prototypes that can be quickly tested and refined. Once a working prototype is available, usability testing is conducted with the user. The user’s interactions with the prototype provide valuable insights for further product refinement. Involving users at the design stage minimizes the likelihood of developing applications that do not address the users’ needs or are not suitable for the user’s workflow.^27^ Here, we review five publications that used HCAI principles during the design phase of the AI tools.

An example of how AIDTs can be iteratively developed, taking into account user feedback, is CheXplain, an AI-enabled chest X-ray analysis tool.^38^ The authors improved CheXplain by including explanations to support its diagnostic capability. They conducted a survey to understand the types of explanations needed by referring physicians and radiologists. Clinicians indicated that AIDTs must explain themselves with probabilities and professional knowledge. In addition, referring physicians expected the tools to annotate X-ray images. The authors worked with physicians during development and co-designed several low-fidelity prototypes featuring the requested components. These features were then integrated into a high-fidelity prototype, which allowed further improvement to iterations by having physicians interact with the new prototype. The authors proposed recommendations that can improve human–AI interactions in the context of medical AI systems. For instance, they recommended allowing the users to decide how many explanations are presented and how much time they spend trying to understand AI. This could be implemented through a toggle button that adjusts the number of explanations displayed. They also suggested tailoring AI explanations to specific clinical decisions or problems, rather than providing general explanations of the AI. Physicians were found to focus on understanding aspects of AI’s results only when they were relevant to their own hypothesis or differential diagnosis.

Involving end users in defining the problems an AIDT should solve is a crucial step in HCAI and human-centered design. This step was outlined in a report on an AI pipeline for the preoperative differential diagnosis of leiomyosarcoma versus leiomyomas.^41^ The authors created a group of healthcare professionals specialized in gynecology to represent the end users during the design phase. They gathered information about their needs and priorities using semistructured interviews. After developing user interface prototypes, participants were invited to discuss the proposed prototypes and suggest improvements during workshops. The AI system was refined by considering guidelines from People + AI Research (PAIR)^73^ and Human-AI interaction from Microsoft,^74^ focusing primarily on guidelines considering XAI.

Zhang et al.^39^ investigated a new data annotation tool (PathNarratives) for pathologists that included a hierarchical decision-to-reason data structure and a narrative annotation process. The authors consulted the World Health Organization pathological clinical guidelines; analyzed pathology report templates; and observed two pathologists for their diagnosis, browsing, and reasoning. This approach ensured their AIDT was tailored to how pathological decisions are made, explained, and documented. The tool was used to label lesion areas and describe the features of these lesions. They found that the classification performance of the AI improved when the model also included more comprehensive reasoning information. Moreover, detailed explanations increased clinicians’ trust and confidence in AI-assisted diagnosis.

Another example of projects using HCAI during the development of AIDTs is the case of BreastScreening-AI.^40^^,^^42^ The tool allows radiologists to visualize and manipulate images while accessing AI diagnostic recommendations that can be readily accepted or rejected. The authors focused on improving the trust and usability of their AIDT by studying how clinicians accept and use AI tools and how clinicians are affected by AI assistance in different clinical contexts. They found that AI explanations of the classification using heatmaps were associated with increased understanding and trust by clinicians. The study also revealed that clinicians’ variability and time spent on each image were reduced when using the AI assistant, leading to lower mental and physical workloads. In addition, they reviewed the perceived usability of the system, finding that 86% of clinicians agreed that AI assistance would not increase diagnostic complexity, and that 85% preferred working with the AI assistant over working without it.

### Prevalence of the human-centered AI perspective in AI-powered diagnostic tools for skin cancer

From an HCAI perspective, the main aim of AI diagnostic assistants is to empower and enhance the performance of oncologists. Interestingly, the seminal papers that demonstrated the potential of deep neural networks to classify skin lesions often explicitly compared the performance of AI systems and humans as if competing against each other.^1^^,^^4^^,^^5^^,^^48^ In the field of dermatology, phrases such as ‘man against machine,’^4^ ‘deep learning outperformed dermatologists,’^1^^,^^75^ and ‘human against machine datasets’^76^ are common, reflecting this competitive framework. From an HCAI perspective, the critical question is not whether convolutional neural networks outperform dermatologists but whether oncologists perform better with AI diagnostic assistants than when working without such tools.

Our second search investigated the proportion of AI diagnostic assistants for skin cancer developed with a human-enhancement perspective and reviewed the evidence that these tools improve the diagnosis accuracy of dermatologists. We identified 285 publications published between 2019 and 2023 that used AI for skin cancer diagnosis and then narrowed our focus to 37 publications from 2019 and 93 publications from 2023. The analysis revealed that only 3 out of 37 (8.1%) papers in 2019 and 3 out of 93 (3.2%) papers in 2023 assessed the performance of dermatologists with and without AI tools^43^, ^44^, ^45^, ^46^, ^47^, ^48^ (Table 2). More articles compared AI tools with dermatologists (21.6% and 7.5%). From 2019 to 2023, there was a trend toward more papers comparing AI tools with one another without direct comparison with oncologists (from 19 to 66 articles, representing 51.3% and 70.9%, respectively).

We found six research articles (three in 2019 and three in 2023) assessing dermatologists’ performance with and without AI assistance.^43^, ^44^, ^45^, ^46^, ^47^, ^48^ Three additional papers were found using the snowballing technique.^28^^,^^49^^,^^50^ In all instances (9/9 articles), dermatologists’ diagnostic accuracy increased when their decisions were supported by AI. A recent meta-analysis study covering 2017-2022 also confirmed the positive impact of AI assistance on dermatologists’ diagnoses.^6^

In our search, we noted that although most of these studies were retrospective (8/9), the positive impact of AI support has now been observed in a prospective study.^43^ In this study, dermatologists first carried out examinations without AI support, assigning a probability of malignancy for each lesion and proposing a management decision (no action, follow-up examination, or excision). The suspected lesion sites were then analyzed using AI (Moleanalyzer pro, FotoFinder Systems, Bad Birnbach, Germany), and the dermatologists could revise their decisions while considering the AI results. Diagnostic sensitivity and specificity improved when dermatologists integrated AI results into their decision making. Unnecessary excisions of benign nevi were reduced by 19.2% when dermatologists collaborated with the AI system.

### Explainable AI (XAI) brings human–AI interactions into the limelight

There has been a significant push toward using XAI in AIDTs in recent years. From the perspective of end users, XAI can illuminate the otherwise opaque AI decision-making process. As explainability directly affects the user’s trust in AI products,^28^ it is inherently part of HCAI.

We searched for research articles that used XAI in the context of AIDTs for skin cancer to assess how explanations are tailored to dermatologists and determined whether the impact of XAI on dermatologists had been investigated. We found 26 papers that used XAI in the context of AIDTs for skin cancer.^28^^,^^45^^,^^48^^,^^49^^,^^51^, ^52^, ^53^, ^54^, ^55^, ^56^, ^57^, ^58^, ^59^, ^60^, ^61^, ^62^, ^63^, ^64^, ^65^, ^66^, ^67^, ^68^, ^69^, ^70^, ^71^, ^72^ The types of explanations provided to the users varied across these papers. Explanations based on images were the most frequent, appearing in 17 of 26 articles.^28^^,^^49^^,^^51^^,^^52^^,^^54^, ^55^, ^56^, ^57^, ^58^, ^59^^,^^63^, ^64^, ^65^, ^66^, ^67^^,^^70^^,^^72^ Grad-CAM was the most commonly used image-based technique, featuring in seven articles.^28^^,^^51^^,^^55^^,^^57^^,^^58^^,^^65^^,^^72^ Textual explanations based on concepts familiar to dermatologists were used in 8 of 27 articles.^28^^,^^52^^,^^54^^,^^55^^,^^66^^,^^69^^,^^71^^,^^72^ In this section, we focused on research articles that studied human–XAI interactions, namely, the explanation preferred by dermatologists,^57^ the use of domain-specific concepts as explanations,^28^^,^^66^^,^^69^^,^^72^ and the impact of XAI on the dermatologist’s perception of AI tools.^28^

One key question when implementing XAI algorithms for AIDTs is which explanations should be included. Giavina-Bianchi et al.^57^ compared five visual explanation techniques for melanoma classifications (Grad-CAM, Grad-CAM++, Eigen-CAM, Score-CAM, and LIME). They evaluated how well the regions highlighted by these XAI techniques overlapped with areas identified by two dermatologists as clinically relevant based on asymmetry, border irregularity, and color heterogeneity. The agreement scores were the highest for Grad-CAM, followed by Grad-CAM++ and LIME. In a qualitative assessment of the five methods, the dermatologists preferred Grad-CAM and Grad-CAM++ over other techniques.

Several research groups have worked on models that use dermatological concepts to explain the diagnosis of AIDTs. The main advantage of using clinically relevant dermatological concepts is that dermatologists already use them when making diagnoses without assistance. Dermatologists could potentially trust AI-supported diagnosis further if AIDTs use similar concepts to reach their predicted diagnosis.^28^

Barata et al.^66^ developed a hierarchical classifier that mimicked the thought process of dermatologists. The classification was achieved in three steps using a long short-term memory model, allowing a previous prediction to influence the next. The lesions were classified based on their origin (melanocytic or nonmelanocytic), severity (benign or malignant), and finally, a differential diagnosis (e.g. atypical nevi, melanoma, basal cell carcinoma). The model achieved competitive scores on dermoscopic images.

Another approach involves mapping human-understandable concepts within the latent representation of neural networks, known as concept activation vectors.^69^^,^^77^ In dermatological images, one can assess the contribution of standardized dermatology concepts,^78^ including *Pigment Networks*, *Streaks*, *Regression Structures*, and *Blue-Whitish Veils,* to the decision made by neural networks.^69^

A third approach is to use convolutional neural networks to predict the dermatological concepts in skin images and base the predicted diagnosis on these concepts.^28^^,^^72^ An advantage of these models is that a saliency map could be generated for each dermatological concept. These models achieved a diagnostic performance on par with control classification-only models.^28^^,^^72^ One limitation of using the dermatologist’s vocabulary to explain the predicted diagnosis is that model training relies on supervised learning, where experts annotate hundreds of images with selected concepts. The success of this approach depends on the quality and quantity of annotated datasets.

The impact of XAI on dermatologists has been recently assessed in a retrospective study.^28^ The XAI model predicted dermatological concepts describing the lesions and a diagnosis based on the concepts. The diagnosis accuracy of dermatologists was assessed without AI, with AI, or with XAI. The results showed that the accuracy of dermatologists increased when using AI, but providing additional lesion characteristics did not improve accuracy further. Nevertheless, the clinicians’ confidence in their diagnosis increased when the AI provided a list of lesion characteristics associated with the diagnosis. Similarly, XAI’s explanations increased the trust in the AI’s decisions compared with an AI providing a diagnosis without explanation.

## Discussion

This systematic review aimed to assess the application of human-centered AI (HCAI) principles^29^ in developing AIDTs for oncology and exemplifying their use in more detail for skin cancer diagnostics. We carried out three literature searches. We reviewed five publications illustrating how user feedback can be incorporated during the design phase of AIDTs for oncology,^38^, ^39^, ^40^, ^41^, ^42^ increasing the likelihood that AIDTs meet the real-world needs of their users. Zooming in on AIDTs for skin cancer, our review of nine studies revealed robust evidence that AIDTs enhance dermatologists’ decision making.^28^^,^^43^, ^44^, ^45^, ^46^, ^47^, ^48^, ^49^, ^50^ This human-enhancement effect was also observed in clinical settings.^43^ Finally, we found that XAI has become prevalent in AIDTs for skin cancer, with 26 publications on the subject. In four studies, explanations were tailored to the end users via clinically relevant concepts.^28^^,^^66^^,^^69^^,^^72^ One study reported the positive impact of user-tailored XAI on user trust.^28^ These findings underscore the importance of incorporating HCAI principles to develop AIDTs that are not only technically proficient but also user-friendly, capable of improving clinical decision making, and trustworthy.

Clinicians’ acceptance of AIDTs depends on several factors, including how suitable the tool is to the workflow, the time it takes to use, how easy it is to use, and whether the information presented is novel.^27^^,^^79^ To facilitate acceptance of AI among clinicians, it is advisable to integrate end users in the early stages of AI development,^80^ a central concept of human-centered design and HCAI. We found four examples of AIDTs designed and/or developed closely with the end user.^38^, ^39^, ^40^, ^41^, ^42^ Several research strategies were used to gather information about the users, including user surveys on the type of explanations they expect,^38^ semistructured interviews with a group of users,^41^ development of system requirements based on information obtained from the users,^38^^,^^41^ co-design of low-fidelity prototypes with clinicians,^38^ and evaluation of prototypes or early product by users.^38^^,^^41^^,^^42^ Recommendations were also put forward based on user feedback. For instance, making it possible for the user to decide how much explanatory information they receive makes the application more adaptable to the clinician workflow.^38^ Users also preferred clinically relevant explanations rather than more general explanations about AI.^38^ These studies show that user feedback can guide the development of AIDTs that are better adapted to their future users.

HCAI places an important emphasis on AI systems that augment human abilities instead of replacing humans. We noted that several earlier studies on AIDTs for skin cancer often framed AIDTs as competitors rather than assistants of dermatologists. Fortunately, several studies now directly test whether AIDTs can improve dermatologists’ performance. Of the nine studies we reviewed, all showed that dermatologists’ accuracy was higher when assisted by AI. Importantly, this improvement has also been observed in clinical settings (*n* = 1). Although the effect of AIDTs on clinicians’ performance in clinical settings will have to be replicated, such observations are critically important for the adoption of AIDTs in clinics, as perceived usefulness is a key factor influencing the acceptance of new technology.^79^

We observed that most studies on AIDTs did not directly involve clinicians but instead compared different AI models against each other. Although this approach is less central to HCAI principles, developing new models and algorithms with high accuracy is critical, as such models can ultimately be tested as AI assistants for clinicians.

XAI has become a common aspect of AI diagnostic assistants in oncology, partly due to regulatory frameworks such as the European Union General Data Protection Regulation and the AI Act, which mandate that AI-based decisions be explainable.^33^^,^^35^ Here, we reviewed several studies on XAI that tailored their AI explanations to the user.^28^^,^^66^^,^^69^^,^^72^ For instance, in the context of skin cancer, using explanations that are based on common dermatological knowledge provides indications to dermatologists that the AIDT uses information that they commonly use when making a diagnosis. This approach also leads to clinically relevant explanations, which users prefer over general explanations of AI.^38^ This type of explanation has been shown to increase the trust of clinicians in their diagnosis and their trust in AI products.^28^ These two factors could contribute to the adoption of AIDTs by clinicians.

The development of AIDTs for oncology using an HCAI perspective remains a relatively new area and there is no established or standard procedure to do so. However, several software companies have proposed guidelines to foster the development of HCAI products or products guided by knowledge of human–AI interactions.^73^^,^^74^^,^^81^^,^^82^ For instance, Microsoft drew a list of 18 guidelines from the academic literature and their experience with AI systems.^74^^,^^81^ These guidelines could guide the development of AIDTs. Alternatively, guidelines to apply human-centered principles to developing XAI models for medical images have also been published.^37^ Future studies will need to test how these guidelines impact AIDT adoption or usability.

One limitation of our review is that HCAI might be used in articles that do not contain our search keywords. For instance, articles focusing on XAI might not explicitly mention the term HCAI, although XAI is one aspect of HCAI.^29^ It is also likely that user studies of AI products approaching commercialization will not be published. Likewise, the iterative refinement of AI products, a component of HCAI, is likely to occur for commercialized systems but is unlikely to be disclosed as research articles.

## Conclusion

In conclusion, this review underscores the significant role of human-centered AI (HCAI) in developing AIDTs for oncology, particularly in skin cancer diagnostics. The integration of user feedback, evidence showing improved clinical decision making, and the utilization of explainable AI (XAI) are pivotal in fostering trust and usability among clinicians. These elements are crucial for the widespread clinical adoption of AIDTs, ultimately enhancing patient outcomes.
